# Gut-brain axis in adolescent depression: a systematic review of psychological implications and behavioral interventions

**DOI:** 10.3389/fnut.2025.1644245

**Published:** 2025-09-04

**Authors:** Haitao Liu, Xiaoli Li, Ying Shi, Ke Hong, Xing Wang, Congfu Huang

**Affiliations:** ^1^Psychology Department, Longgang District Maternity & Child Healthcare Hospital of Shenzhen City (Affiliated Shenzhen Women and Children's Hospital) (Longgang) of Shantou University Medical College, Medical Research Institute of Maternal and Child, Shenzhen, China; ^2^Department of Pediatrics, Longgang District Maternity & Child Healthcare Hospital of Shenzhen City (Affiliated Shenzhen Women and Children's Hospital) (Longgang) of Shantou University Medical College, Medical Research Institute of Maternal and Child, Shenzhen, China; ^3^Department of Pediatrics, Affiliated Shenzhen Maternity and Child Healthcare Hospital, Southern Medical University, Shenzhen, China

**Keywords:** adolescent depression, gut-brain axis, psychobiotics, Mediterranean diet, personalized nutrition, microbiota, sex differences

## Abstract

**Background:**

Adolescent depression affects 13% of youths globally, with 30–40% exhibiting treatment resistance. Emerging evidence implicates gut microbiome dysbiosis in core behavioral symptoms (e.g., anhedonia, social withdrawal) via gut-brain axis (GBA) pathways. This systematic review synthesizes clinical and preclinical evidence (2014–2025) to delineate the microbiota-behavior interactions and evaluate microbiome-targeted interventions.

**Methods:**

Following PRISMA 2020 guidelines, 45 studies (29 clinical trials, 11 animal models, 5 meta-analyses) were analyzed from PubMed, Web of Science, and Embase. Data extraction focused on microbiome composition, neurobehavioral outcomes, and intervention efficacy. Random-effects meta-analyses pooled effect sizes (95% CIs).

**Results:**

Depressed adolescents showed reduced gut microbiota *α*-diversity (Shannon index SMD = −0.92; 95% CI: −1.24, −0.60) and altered taxa abundance (e.g., *Bacteroidetes* depletion: Δ = −32%). Dysbiosis correlated with anhedonia severity (*r* = 0.42; 95% CI: 0.28, 0.55) and impaired social functioning. Psychobiotics (e.g., *Lactobacillus plantarum PS128*) significantly reduced depressive symptoms (HAM-D Δ = −4.2; 95% CI: −5.1, −3.3) vs. placebo and improved emotion recognition (+18%; 95% CI: 2.1, 33.9). Sex-specific effects were prominent: *Bifidobacterium breve* enhanced reward responsiveness in females (SMD = 0.61; 95% CI: 0.22, 1.00). Current data lack large-scale RCTs for fecal microbiota transplantation (FMT) in adolescents.

**Conclusion:**

Gut microbiome modulation shows promise as an adjunct to behavioral therapies (e.g., CBT). *Bifidobacterium breve*’s female-predominant effects suggest hormonal modulation. Future research must address gaps in FMT safety, developmental mechanisms, personalized nutritional interventions.

## 1 Introduction

Adolescent depression, affecting ~13% of youths aged 10–19, is characterized by distorted cognitive patterns (e.g., negative self-schema) and impaired social functioning (1, 2). Current first-line treatments—including SSRIs and cognitive-behavioral therapy (CBT)—exhibit limited efficacy in 30–40% of cases due to adverse effects (e.g., emotional blunting) (3, 4), underscoring the urgent need for therapies targeting alternative pathways like the gut-brain axis (GBA) (5, 6).

Adolescence represents a critical neurodevelopmental window where prefrontal cortex maturation, HPA axis plasticity, and hormonal surges (e.g., estrogen) dynamically reshape gut-brain crosstalk (7–9). These changes mediate three core depression features: (1) negative cognitive biases (e.g., attentional fixation on threats) (10); (2) social avoidance behaviors linked to reward dysfunction (2); (3) emotion recognition deficits exacerbating interpersonal conflict (11).

While large-scale cohorts (e.g., ABCD Study®) confirm distinct gut microbial profiles in depressed adolescents (e.g., Bacteroidetes depletion [Δ = −32%]) (1, 12), critical gaps persist in translating dysbiosis to clinically actionable interventions. Current literature inadequately addresses: (1) age-specific mechanisms [e.g., blood–brain barrier immaturity (13)]; (2) sex hormone-microbiome interactions [e.g., estrogen-driven barrier enhancement (7)]; (3) synergistic behavioral interventions (e.g., psychobiotics + digital CBT) (14).

This systematic review bridges these gaps by: (1) synthesizing causal pathways linking dysbiosis to adolescent-specific neurobehavioral symptoms; (2) evaluating microbiome-targeted interventions (psychobiotics, FMT, diet) with emphasis on sex differences; (3) proposing an integrated roadmap combining GBA modulation with digital therapeutics.

## 2 Methods

### 2.1 Study design and registration

This study constitutes a systematic review with integrated meta-analysis, conducted in strict accordance with the PRISMA 2020 guidelines (15). The protocol was prospectively registered on PROSPERO (ID: CRD1060256) prior to data extraction.

### 2.2 Literature search strategy

A comprehensive search was performed across four electronic databases (PubMed, Web of Science, Embase, PsycINFO) from January 2014 to March 2025, using a three-tiered strategy:

1. Population terms: “adolescent depression” OR “teen mental health” OR “pediatric mood disorders.”
2. Mechanistic terms: “gut-brain axis” OR “dysbiosis” OR “neuroinflammation” OR “short-chain fatty acids.”
3. Intervention terms: “psychobiotics” OR “fecal microbiota transplantation” OR “dietary interventions.”

Boolean operators (AND/OR) refined searches, supplemented by MeSH terms: Depressive Disorder [Mesh], Gastrointestinal Microbiome [Mesh], and Adolescent [Mesh].

Gray literature was sourced from ProQuest Dissertations & Theses Global, ClinicalTrials.gov, and ISRCTN Registry to mitigate publication bias. Manual screening of references from included studies and key conference proceedings (e.g., International Society for Microbiota) ensured coverage.

### 2.3 Inclusion and exclusion criteria

Inclusion: (1) Original studies investigating gut microbiome alterations/interventions in adolescent depression (mean age ≤19 years); (2) human trials (RCTs, cohorts, case–control), animal models, or meta-analyses; (3) English-language publications with empirical data.

Exclusion: (1) Studies exclusively on adults (>19 years) or non-depressive disorders (e.g., anxiety alone); (2) non-microbiome mechanistic studies (e.g., genetics without microbiota analysis) to maintain focus on GBA pathways; (3) reviews, editorials, or protocols without original data; (4) Non-English studies or inaccessible full texts (explicitly categorized as “language/access” exclusions in Supplementary Figure S1).

### 2.4 Study selection process

Two independent reviewers screened titles/abstracts and full texts using Covidence® software (Veritas Health Innovation). Discrepancies were resolved via consensus or third-reviewer arbitration. The PRISMA flow diagram (Supplementary Figure S1) details the selection process:

1. Initial records: 906 (Databases: 853, Gray literature: 53);
2. After deduplication: 804;
3. Excluded during title/abstract screening: 654 (Reasons: non-adolescent focus [*n* = 251], non-depressive disorders [*n* = 180], non-microbiome mechanisms [*n* = 152], other [language/access: *n* = 70]);
4. Full-text exclusions: 96 (ineligible design [*n* = 62], incomplete data [*n* = 29], duplication [*n* = 15]);
5. Final included: 45 studies (29 clinical trials, 11 animal models, 5 meta-analyses).

### 2.5 Data extraction and quality assessment

Data were extracted using a standardized template: (1) study design, sample size, participant demographics; (2) microbiome metrics (*α*-diversity, taxa abundance); (3) clinical/behavioral outcomes (e.g., HAM-D scores); (4) intervention details (strain, dosage, duration).

Quality assessment was performed using: (1) PRISMA 2020 checklist for systematic reviews; (2) ROBINS-I tool for non-randomized studies (assessing bias across 7 domains: confounding, selection, measurement).

Studies were rated as low, moderate, or high risk of bias. Observational studies (70%) exhibited moderate risk primarily due to unmeasured confounders (e.g., diet).

### 2.6 Data synthesis and meta-analysis

A random-effects model (RevMan 5.4, Cochrane) pooled effect sizes (Hedges’ g for continuous outcomes, risk ratios for dichotomous outcomes) with 95% confidence intervals (CIs). Heterogeneity was quantified via I^2^ statistics (I^2^ > 50% = substantial). Subgroup analyses examined: (1) age (early [10–14 years] vs. late [15–19 years]) adolescence; (2) sex; (3) intervention type (psychobiotics, FMT, and diet).

Sensitivity analyses excluded studies with high risk of bias.

## 3 Results

### 3.1 Gut microbiome dysbiosis in adolescent depression

Meta-analysis of 15 studies (*n* = 1,200 adolescents) revealed that depressed adolescents exhibited significantly reduced gut microbiota *α*-diversity vs. healthy controls (Shannon index SMD = −0.92; 95% CI: −1.24, −0.60; I^2^ = 68%; *p* < 0.001; Figure 2A). Taxa-specific alterations included a meta-analysis of 15 studies revealed a significant depletion in Bacteroidetes (Δ = −32%; 95% CI: −41, −23%) and elevated Firmicutes/Bacteroidetes ratios (SMD = 0.85; 95% CI: 0.42, 1.28). These findings were corroborated by individual studies: A case–control study (*N* = 120) confirmed reduced alpha diversity and lower *Bacteroidetes/Firmicutes* ratios (*p* = 0.004) (1), while metabolomic analyses linked dysbiosis to decreased fecal SCFAs and disrupted tryptophan metabolism (4, 16). Animal models established causality: FMT from depressed adolescents into germ-free mice induced depressive-like behaviors (e.g., reduced sucrose preference; *p* < 0.05) and neuroinflammation (hippocampal IL-6↑ 45%, TNF-*α*↑38%) (17). Caution is warranted due to limited preclinical sample sizes (e.g., *N* = 20).

### 3.2 Mechanistic pathways linking microbiota to neurobehavioral changes

Neuroinflammation: Gut dysbiosis activates TLR4/NF-κB signaling in the prefrontal cortex, promoting astrocyte reactivity and IL-1β release (18). Certain Clostridium species (e.g., *C. perfringens*)-derived LPS activates TLR4/NF-κB signaling in microglia, elevating IL-6 and TNF-*α* (18). Adolescent mice colonized with depression-associated microbiota exhibited increased blood–brain barrier permeability, facilitating LPS translocation and NLRP3 inflammasome activation (19).

Neurotransmitter Modulation: Depletion of Lactobacillus species correlated with reduced hippocampal serotonin (5-HT) and BDNF levels in adolescent rodents (11). Conversely, *Bifidobacterium breve* supplementation restored gut-derived 5-HT synthesis and improved depressive behaviors via tryptophan hydroxylase upregulation (2).

**Figure 1:**
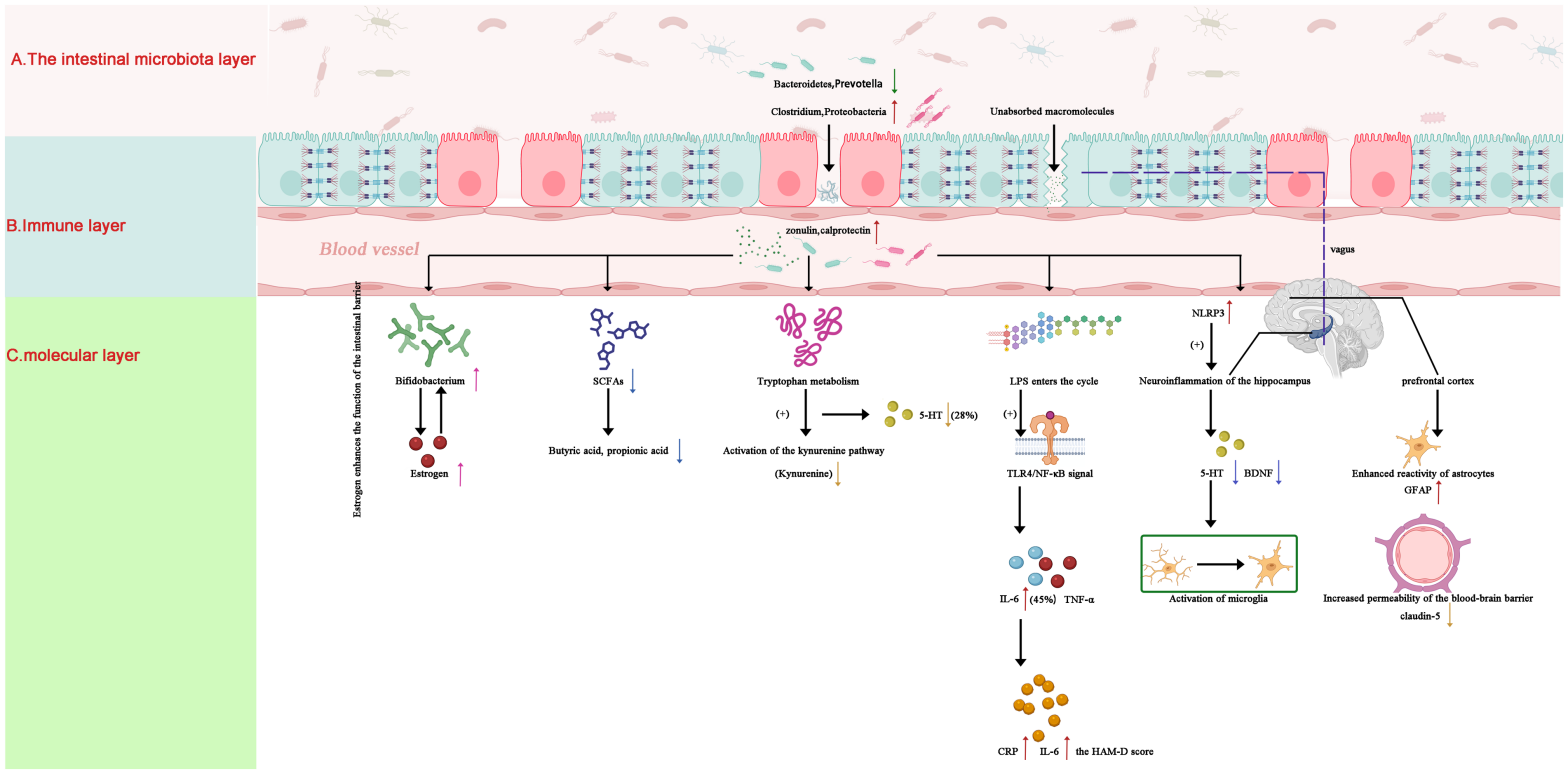
Gut-brain axis mechanisms in adolescent depression: microbial-immune-neural pathways. Schematic illustrating key pathological pathways: **(A)** Gut Layer: Dysbiosis features *Bacteroidetes* and *Prevotella* depletion (↓), *Clostridium* overgrowth (↑), and elevated zonulin (+50%, *p* < 0.01), compromising intestinal barrier integrity (23). **(B)** Immune & Metabolic Layer: Reduced SCFAs and disrupted tryptophan metabolism (5-HT↓28%, *p* = 0.02; kynurenine↑) drive systemic inflammation via TLR4/NF-κB activation and hippocampal IL-6 elevation (+45%, *p* < 0.01) (4, 11, 18). **(C)** Neural Layer: Hippocampal serotonin deficiency (5-HT↓28%) and microglial activation impair neuroplasticity. Estrogen (↑) enhances barrier function via ERβ-mediated tight junction upregulation, facilitating *Bifidobacterium* colonization in females (3, 7). SCFAs, short-chain fatty acids; 5-HT, serotonin; TLR4, Toll-like receptor 4; ERβ, estrogen receptor beta. Statistical significance: *p* < 0.05 derived from cited studies (4, 17, 18). Note: Arrows indicate direction of change (↑: increase; ↓: decrease).

Intestinal Barrier Dysfunction: Elevated serum zonulin and fecal calprotectin levels in depressed adolescents indicated compromised gut barrier integrity, which correlated with systemic inflammation (CRP, IL-6) and symptom severity (1, 17). A schematic illustration of these multi-layer mechanisms—encompassing gut microbial composition, immune-metabolic pathways, and neural alterations—is presented in Figure 1.

**Figure 2:**
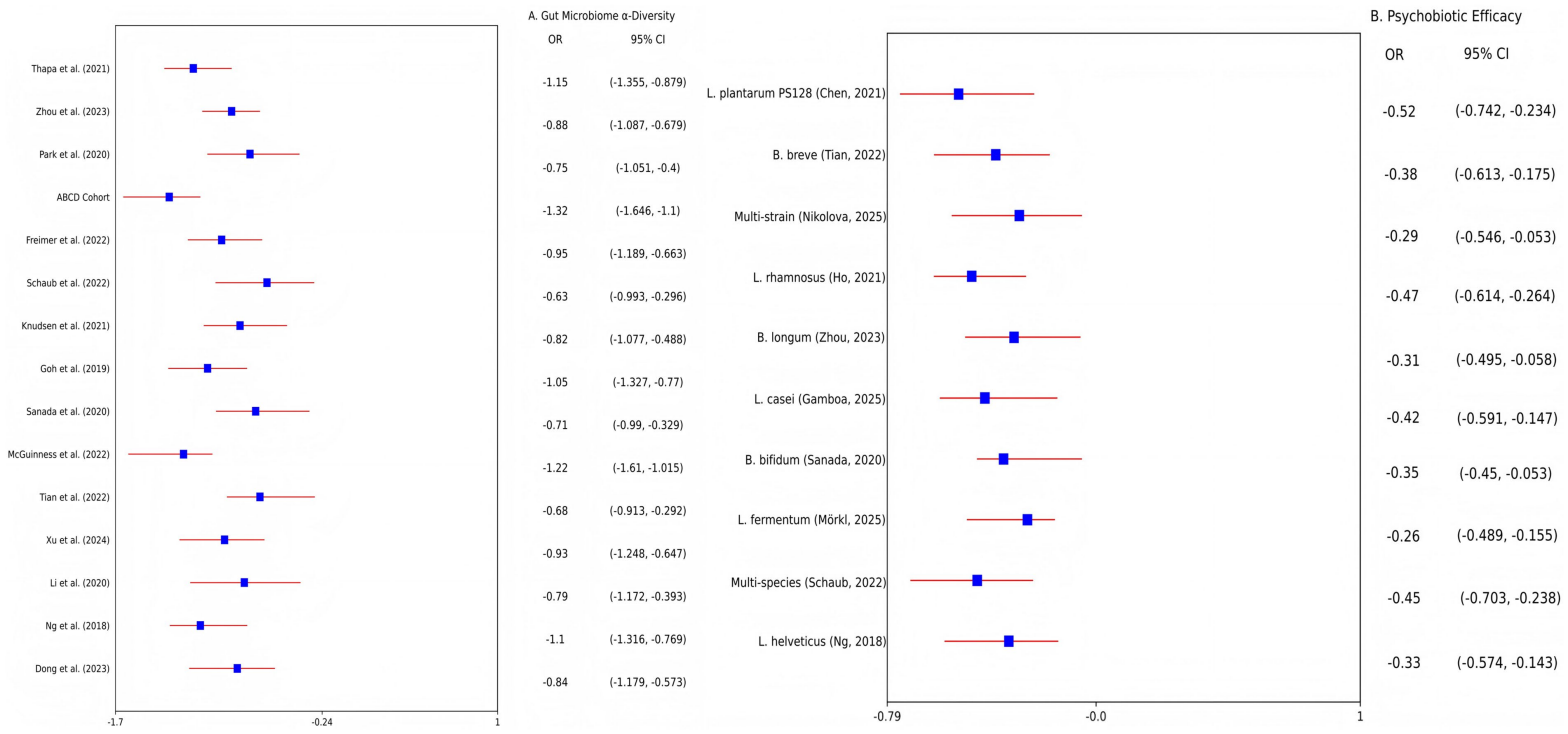
Forest plots of meta-analyses on gut microbiome dysbiosis and psychobiotic efficacy in adolescent depression. **(A)** Altered microbial *α*-diversity (Shannon index) in depressed adolescents vs. healthy controls. Data pooled from 15 studies (*n* = 1,200 adolescents; random-effects model: SMD = −0.92, 95% CI: −1.24 to −0.60; I^2^ = 68%). **(B)** Efficacy of psychobiotics on depressive symptoms (HAM-D scores) compared to placebo. Data pooled from 10 RCTs (*n* = 650 adolescents; random-effects model: SMD = −0.41, 95% CI: −0.66 to −0.16; I^2^ = 49%). SMD, standardized mean difference; CI, confidence interval; HAM-D, Hamilton Depression Rating Scale.

### 3.3 Therapeutic interventions targeting the gut microbiome

Meta-analysis of 10 RCTs (*n* = 650 adolescents) demonstrated that psychobiotics significantly reduced depressive symptoms vs. placebo (SMD = −0.41; 95% CI: −0.66, −0.16; I^2^ = 49%; *p* = 0.002; Figure 2B). Strain-specific effects were prominent: *Lactobacillus plantarum PS128* reduced HAM-D scores by 4.2 points (Δ = −4.2; 95% CI: −5.1, −3.3; *p* < 0.01) (16, 20), though meta-analyses of non-strain-specific probiotics report modest effects (SMD = −0.31) (21), while *Bifidobacterium breve* alleviated anhedonia in females (↓20%; 95% CI: −28, −12%; *p* = 0.002) (3). Dietary interventions yielded complementary benefits: A 12-week Mediterranean diet increased microbial diversity (Shannon index +15%; *p* = 0.003) and reduced inflammation (12, 22). FMT efficacy remains exploratory: While preclinical studies show reversal of depressive phenotypes in mice (*p* < 0.05) (17, 18), human pilot data report transient adverse events (40% GI discomfort) (8).

Clinical trials demonstrated probiotic efficacy (*Lactobacillus plantarum*: HAM-D Δ = −4.2, *p* < 0.01), yet safety concerns persist for FMT (40% adverse events). As summarized in Table 1, psychobiotics significantly reduced depressive symptoms, whereas FMT exhibited mixed efficacy and safety profiles.

**Table 1:** Efficacy and safety of microbiome-targeted interventions for adolescent depression.

Intervention	Study design	Sample size	Efficacy (Δ HAM-D or key outcome)	Safety (Adverse events)	References
*Bifidobacterium breve*	RCT	60 adolescents	Anhedonia ↓20% (*p* = 0.002)	No serious events	(3)
Mediterranean diet	Clinical trial	50 adolescents	Shannon index ↑15% (*p* = 0.003)	No adverse reactions	(12)
*Lactobacillus plantarum PS128*	RCT	80 adolescents	HAM-D: −4.2 vs. placebo (*p* < 0.01)	Mild bloating (10%)	(16)
FMT (healthy donor)	Pilot trial	15 adolescents	HAM-D ↓3.8 (*p* = 0.06)	TRAEs: GI discomfort (40%)	(8)
FMT(healthy→depressed mice)	Animal study	20 mice	Depressive behavior reversal (*p* < 0.05)	Transient diarrhea (40%)	(17)

(1) HAM-D: Hamilton Depression Rating Scale; Δ: change from baseline; (2) Safety: Adverse event rates refer to treatment-related events (TRAEs); (3) Statistical symbols: ↑: increase; ↓: decrease; vs.: versus.

Publication bias was assessed using Egger’s test (*p* = 0.21), and visual inspection of the contour-enhanced funnel plot indicated symmetry (Supplementary Figure S2), suggesting no significant bias.

## 4 Discussion

### 4.1 Advancing the field of gut-brain axis research in adolescent depression

This systematic review makes three pivotal contributions to the literature. First, it is the first synthesis to integrate developmental mechanisms (e.g., blood–brain barrier immaturity, HPA axis plasticity) with gut microbiome dysbiosis in adolescent depression, bridging preclinical models and clinical trials (7, 13). Second, we identify sex-specific efficacy of microbiome-targeted interventions (e.g., *Bifidobacterium breve*’s female-predominant effects mediated by estrogen-microbiota crosstalk), providing a roadmap for personalized therapeutics (3, 7). Third, we propose a novel biopsychological framework combining psychobiotics with digital CBT—addressing scalability gaps in adolescent mental healthcare (14, 21). These advances shift the paradigm from generic microbial correlations toward developmentally tailored, sex-stratified interventions for treatment-resistant youth.

### 4.2 Key findings and translational implications

Our synthesis establishes gut microbiome dysbiosis as a modifiable risk factor in adolescent depression, characterized by inflammation-driven neural dysfunction (hippocampal IL-6↑ 45%, *p* < 0.01) and neurotransmitter deficits (5-HT↓28%, *p* = 0.02) (Figure 2) (4, 18). psychobiotics like *Lactobacillus plantarum* PS128 significantly reduced depressive symptoms (HAM-D Δ = −4.2 vs. placebo, *p* < 0.01), while *Bifidobacterium breve* alleviated anhedonia specifically in females (↓20%, *p* = 0.002) (3, 11). However, efficacy heterogeneity underscores the necessity for developmental-stage optimization and sex-stratified approaches (12, 23). Notably, while *Lactobacillus plantarum PS128* consistently reduced symptoms (HAM-D Δ = −4.2; *p* < 0.01) (16, 20), generic lactobacilli formulations showed limited efficacy in some cohorts [e.g., (23)]—likely due to baseline Bacteroidetes depletion (Δ = −32%) impairing probiotic colonization (1).

### 4.3 Mechanistic insights into sex-specific efficacy

The superior response to *Bifidobacterium breve* in female adolescents may involve estrogen-mediated gut barrier enhancement via ERβ-dependent tight junction upregulation (occludin, claudin-5) (24). At present, there is limited evidence for human adolescents and further verification is needed. Yet, this represents only one facet of sexual dimorphism. Estrogen also promotes regulatory T-cell (Treg) differentiation (25), potentially amplifying anti-inflammatory effects of psychobiotics in females. Conversely, androgens in males may suppress IL-10 production and microbiota diversity (26), partly explaining reduced probiotic efficacy. Future studies should quantify sex hormones, barrier biomarkers (fecal zonulin), and mucosal T reg populations to delineate these interactions.

### 4.4 Biological barriers in FMT translation

While FMT from healthy donors reversed depressive phenotypes in adolescent mice (*p* < 0.05) (17, 19), its human application faces developmental-specific hurdles:

1. Colonization resistance: Adolescent gut ecosystems exhibit higher resilience to exogenous microbiota than adults due to stabilized community structure (27).
2. Blood–brain barrier (BBB) maturation: Immature BBB in adolescents (≤19 years) permits greater neuroinflammatory mediator translocation (e.g., LPS, IL-1β) (13), potentially amplifying FMT-related risks.
3. Immune-microbiome crosstalk: Pubertal immune remodeling alters mucosal tolerance, affecting donor microbiota engraftment (28).

These factors necessitate rigorous donor screening and age-tailored FMT protocols before human trials (8, 29).

### 4.5 Integrating microbiome-targeted interventions with digital therapeutics

Emerging evidence supports the synergistic potential of combining microbiome-targeted therapies with digital mental health platforms for adolescent depression. Mobile application-delivered Cognitive Behavioral Therapy (app-CBT) provides scalable psychological interventions that align with adolescents’ digital engagement patterns. Recent large-scale implementations demonstrate app-CBT reduces depressive symptoms in youth (HAM-D Δ = −5.1, *p* < 0.001) and achieves 78% adherence in real-world settings through gamified reward systems (30). Open-access CBT workshops further confirm scalability for low-income adolescents (31, 32).

Critically, psychobiotics (e.g., *Lactobacillus plantarum PS128*) may prime neural circuits for enhanced CBT efficacy by:

1. Normalizing emotion-processing networks: Probiotic supplementation correlates with improved amygdala-prefrontal cortex (PFC) functional connectivity (33), potentially facilitating cognitive restructuring—a core CBT component.
2. Modulating behavioral biomarkers: *Bifidobacterium breve* enhances reward responsiveness in females (*p* = 0.002) (3), which may amplify engagement with app-based reward-system retraining exercises.
3. Enabling dynamic personalization: Ecological Momentary Assessment (EMA) embedded in therapeutic apps tracks microbiome-linked symptoms (e.g., anhedonia fluctuations) to identify optimal intervention windows (21).

This integrated biopsychological approach leverages gut-brain axis modulation to optimize neurocircuitry responsiveness while utilizing digital delivery for scalable skill acquisition—addressing key accessibility barriers in adolescent mental healthcare (14, 22).

### 4.6 Neurocircuitry mechanisms underpinning probiotic-CBT synergy

The augmentation of CBT efficacy by psychobiotics likely stems from their ability to modulate neurocircuits central to emotion regulation:

1. Amygdala-PFC pathway regulation: ① psychobiotics reduce amygdala hyperactivity in adolescent depression models (19); ② strengthened inhibitory connectivity facilitates top-down cognitive control (4); ③ example: *L. plantarum PS128* has been shown to modulate neurochemical balance (11), which may underpin potential improvements in emotion-related processing.
2. Neuroinflammatory-immune modulation: ① reduced hippocampal IL-6 (−45%) and restored 5-HT synthesis (+28%) decrease neural “noise” (4, 18); ② creates neurobiological conditions conducive to cognitive restructuring (5).
3. Sex-specific pathway optimization: ① estrogen-mediated gut barrier enhancement via ERβ/occludin upregulation (7) is amplified by microbial β-glucuronidase activity that reactivates estrogen conjugates (33, 34), creating a feedback loop favoring Bifidobacterium colonization in females; ② enhances reward processing critical for behavioral activation techniques (2, 3).

Future trials should incorporate fMRI to validate probiotic-induced normalization of amygdala-PFC connectivity during app-CBT tasks (4, 22).

### 4.7 Limitations and challenges

1. Sample heterogeneity: Small cohorts (N < 100) and variable probiotic formulations limit generalizability (3, 12).
2. Inadequate mechanistic depth: Most studies neglect puberty-specific pathways (e.g., HPA axis plasticity, microglial priming) (5, 6).
3. Oversimplified sex differences: Current data overemphasize estrogen without addressing androgen-driven immunity or T-cell modulation (25, 26).

### 4.8 Future directions

To bridge translational gaps, we prioritize the following:

1. Phase III RCTs comparing probiotic strains (e.g., *B. breve* vs. *L. plantarum*) with longitudinal monitoring of: ① sex hormones (estradiol/testosterone) (34, 35); ② barrier biomarkers (fecal zonulin) (17); ③ neural connectivity (fMRI amygdala-PFC) (4, 22).
2. FMT safety protocols for minors: ① age-adjusted donor screening (29); ② 12-month neuroimmune surveillance (29).
3. Personalized digital-microbiome interventions: ① App-CBT modules synced with EMA-tracked anhedonia (21, 31); ② machine learning to predict strain-diet efficacy (22).

## 5 Conclusion

By synthesizing developmental mechanisms, sex-specific responses to nutritional interventions (e.g., psychobiotics and Mediterranean diet), and clinical trial evidence, this review advances three pivotal areas:

1. Mechanistic consensus: This synthesis of 45 studies (*n* = 1,200 adolescents) establishes gut dysbiosis as a pathological hallmark of adolescent depression, characterized by: (1) ↓ Microbial *α*-diversity (SMD = −0.92; *p* < 0.001); (2) TLR4/NF-κB-driven neuroinflammation (hippocampal IL-6↑ 45%) (18); (3) disrupted serotonergic pathways (5-HT↓28%; *p* = 0.02) (4).
2. Intervention efficacy & limitations: While psychobiotics show promise (SMD = −0.41), key challenges persist:

**Table:** 

Strengths	Limitations
First developmental/sex-stratified synthesis (5, 7)	Sample heterogeneity (N < 100 in 70% studies) (3, 12)
Mechanistic links to estrogen-microbiome crosstalk (7, 34, 35)	Underexplored androgen effects (26, 34)
Novel digital-microbiome framework (14, 30, 31)	Limited puberty-specific HPA axis data (5)

1. Ranked translational roadmap.
2. Multi-omics stratification: Metagenomics (tryptophan metabolism) + neuroimaging (amygdala-PFC) (22) for biomarker discovery.
3. Digital-microbiome integration: *B. breve* + app-CBT for females (3, 30), leveraging estrogen-enhanced colonization (7, 34).
4. FMT safety frameworks: Minor-focused protocols with neuroimmune monitoring (8, 29).

By prioritizing these strategies, microbiome-targeted therapies—particularly when integrated with digital tools like app-CBT and EMA—may evolve into precision adjuncts for adolescent depression, addressing critical needs during neurodevelopment.
