# Supplementary material for: Gut-brain axis in adolescent depression: a systematic review of psychological implications and behavioral interventions
Source: Front Nutr. 2025 Sep 4;12:1644245. doi: 10.3389/fnut.2025.1644245 (PMC12443687; doi:10.3389/fnut.2025.1644245)
Supplement: Supplementary file 1 [file Table_1.docx]

Supplementary Table S1. Clinical and Preclinical Studies on Gut Microbiome in Adolescent Depression

| **Author (Year)** | **Study Type** | **Sample Characteristics** | **Key**  **Findings** | **Main Methods** | **Conclusion** |
| --- | --- | --- | --- | --- | --- |
| Human Studies | | | | | |
| Park et al. (2020) [12] | Human | N=50, 14–19 years | 16S rRNA sequencing, inflammatory markers | Mediterranean diet ↑ Shannon index (+15%, *p*=0.003) ; inflammation ↓ | Dietary intervention reduces inflammation by regulating microbiota |
| Ho et al. (2021) [13] | Human (RCT) | N=80, 12–19 years | Fecal microbiota analysis, HAM-D | *Lactobacillus plantarum PS128* reduces HAM-D (Δ=−4.2, *p*<0.01) | Specific probiotics alleviate depression |
| Thapa et al. (2021) [1] | Human | N=120, 10–19 years | 16S sequencing, metabolomics | *Bacteroidetes* depletion (Δ=−32%, *p*=0.004); SCFAs ↓; inflammation ↑ | Microbial metabolic disorders correlate with depression severity |
| Tian et al. (2022) [3] | Human (RCT) | N=60, 15–18 years | Metabolomics, behavioral evaluation | *Bifidobacterium breve* ↑ tryptophan metabolism; HAM-D Δ=−4.9 (Δ=−20%, *p*=0.002) | Psychobiotics improve depressive symptoms |
| Animal Studies | | | | | |
| McGuinness et al. (2022) [5] | Meta-  Analysis | 15 studies (human/animal) | Systematic  review | Microbiota diversity ↓; *Bacteroidetes* ↓/*Clostridium* ↑ | Dysbiosis is a biomarker for adolescent depression |
| Zhou et al. (2023) [4] | Animal | Rats, N=30 (adolescent stress) | Tryptophan metabolism, behavioral tests | Microbiota imbalance ↓ hippocampal 5-HT (−28%,*p*=0.02); psychobiotics restore tryptophan hydroxylase | Microbiota regulate neurotransmitter synthesis and behavior |
| Xu et al. (2024) [16] | Animal | Mice, N=20 (adolescence model) | FMT, behavioral tests, neuroinflammation | Healthy-donor FMT reverses depressive behavior (*p*<0.05); hippocampal IL-6 ↓ | FMT improves depression via reduced neuroinflammation |

Notes:

1. Δ: Change from baseline (e.g., HAM-D Δ=−4.2 = 4.2-point reduction).

2. Abbreviations: SCFAs (short-chain fatty acids); 5-HT (serotonin); FMT (fecal microbiota transplantation); HAM-D (Hamilton Depression Rating Scale); RCT (randomized controlled trial).

3. Statistical significance: *p*<0.05.
