# Supplementary material for: Gut-brain axis in adolescent depression: a systematic review of psychological implications and behavioral interventions
Source: Front Nutr. 2025 Sep 4;12:1644245. doi: 10.3389/fnut.2025.1644245 (PMC12443687; doi:10.3389/fnut.2025.1644245)
Supplement: Supplementary file 2 [file Image_1.pdf]

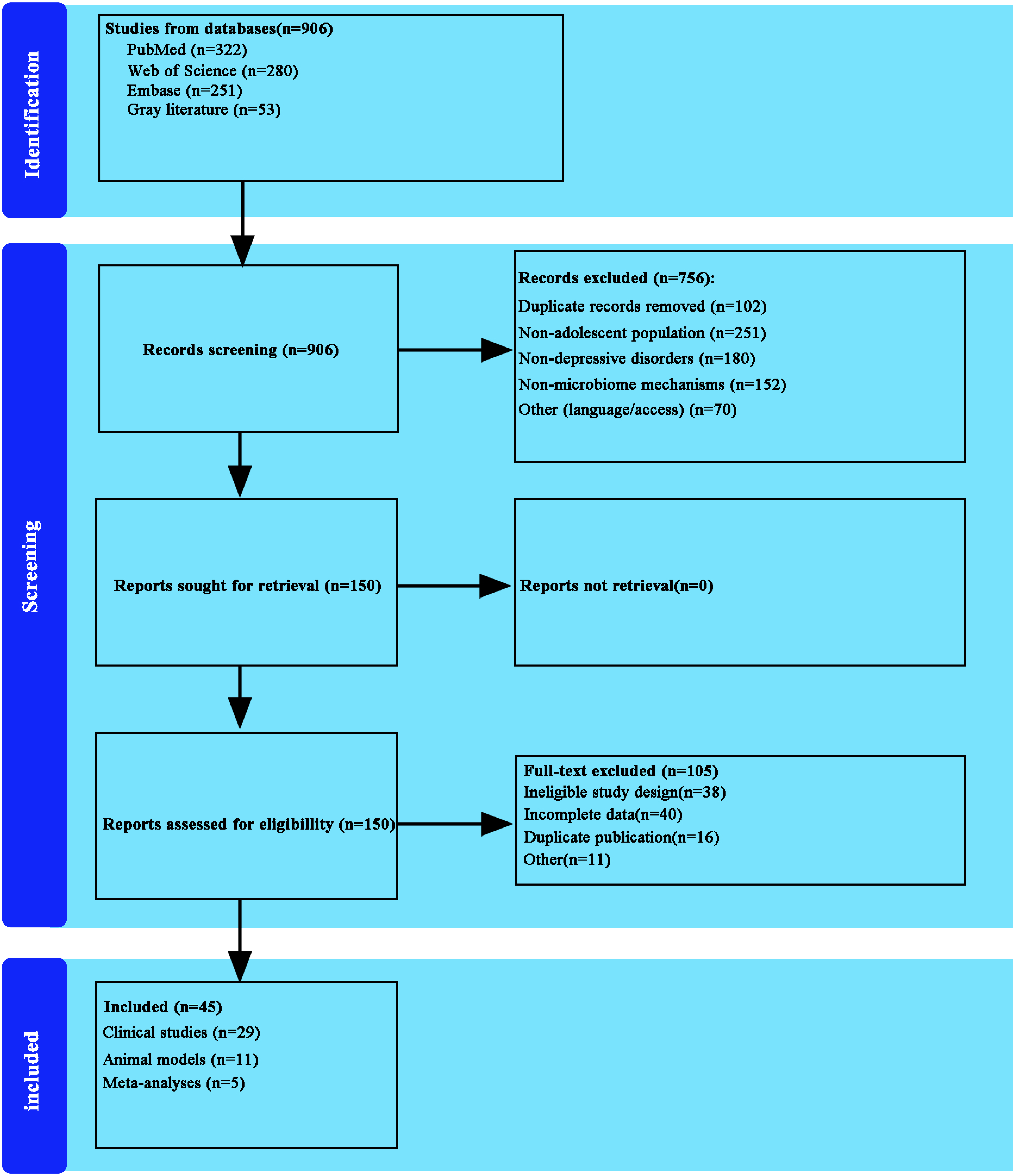

Identification
Studies from databases(n=906)
PubMed (n=322)
Web of Science (n=280)
Embase (n=251)
Gray literature (n=53)
Screening
Records screening (n=906)
Records excluded (n=756):
Duplicate records removed (n=102)
Non-adolescent population (n=251)
Non-depressive disorders (n=180)
Non-microbiome mechanisms (n=152)
Other (language/access) (n=70)
Reports sought for retrieval (n=150)
Reports not retrieval(n=0)
Reports assessed for eligibillity (n=150)
Full-text excluded (n=105)
Ineligible study design(n=38)
Incomplete data(n=40)
Duplicate publication(n=16)
Other(n=11)
included
Included (n=45)
Clinical studies (n=29)
Animal models (n=11)
Meta-analyses (n=5)
