# Supplementary figures and images for: Gut-brain axis in adolescent depression: a systematic review of psychological implications and behavioral interventions
Source: Front Nutr. 2025 Sep 4;12:1644245. doi: 10.3389/fnut.2025.1644245 (PMC12443687; doi:10.3389/fnut.2025.1644245)

Funnel Plot for Psychobiotic Efficacy

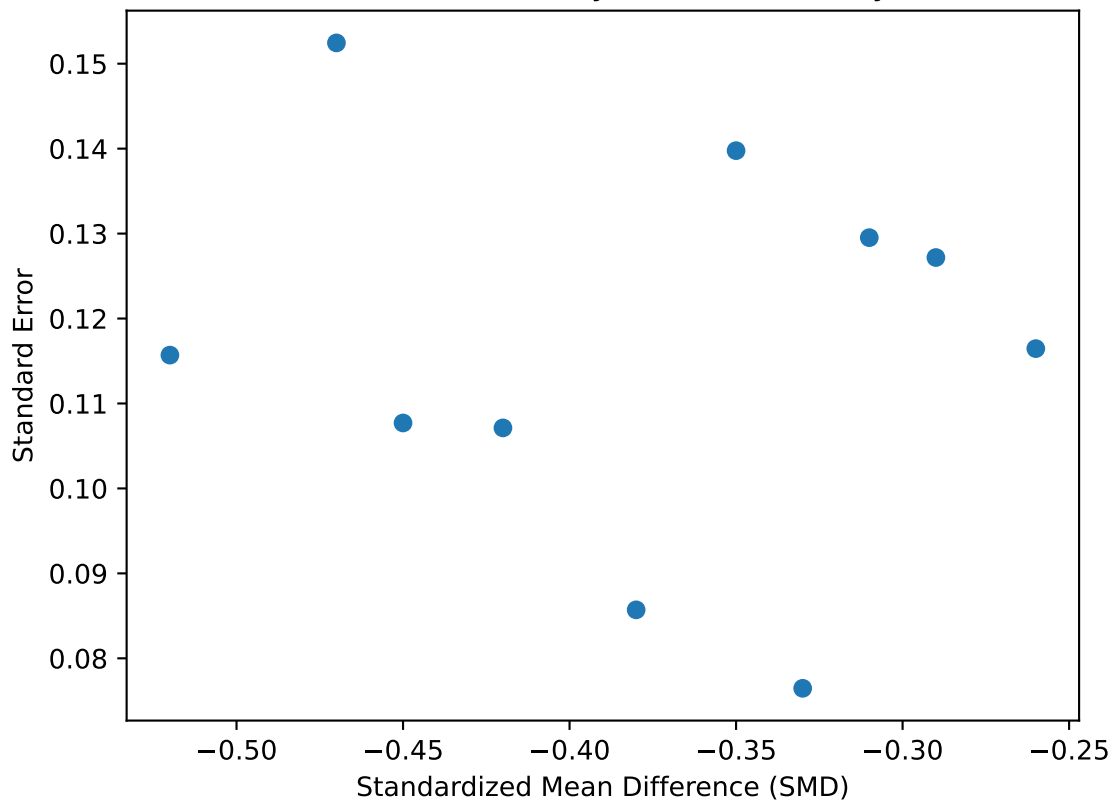

Supplement: Supplementary file 3 [file Image_2.pdf]
